# Why Smoggy Days Suppress Our Mood: Automatic Association Between Clarity and Valence

**DOI:** 10.3389/fpsyg.2019.01580

**Published:** 2019-07-10

**Authors:** Yiguang Liu, Jun Yin, Junying Liang

**Affiliations:** ^1^Department of Linguistics, Zhejiang University, Hangzhou, China; ^2^Department of Psychology, Ningbo University, Ningbo, China; ^3^Center of Group Behavior and Social Psychological Service, Ningbo University, Ningbo, China

**Keywords:** perceptual–conceptual association, clarity, affective valence, embodied cognition, Conceptual Metaphor Theory, Perceptual Symbol Theory

## Abstract

The intuition of clarity–valence association seems to be pervasive in daily life, however, whether there exists a potential association between clarity (i.e., operationalized as visual resolution) and affect in human cognition remains unknown. The present study conducted five experiments, and demonstrated the clarity–valence congruency effect, that is, the evaluations showed performance advantage in the congruent conditions (clear-positive, blurry-negative). Experiments 1 through 3 demonstrated the influence of the perception of clarity on the conceptualization of affective valence, while Experiments 4 and 5 verified the absence of the influence of conceptualization on perception, thus the unidirectionality of clarity–valence association in cognition is confirmed. The findings extend the affective perceptual–conceptual associations into the dimension of clarity, thus providing support for the ideas of embodied cognition as well as implications for our preference for clarity and aversion to blur.

## Introduction

Air pollution is a serious problem globally in the modern society. Suffering from smog, we are prone to feel upset and down not only because of the health hazard but also the blurry view. Our aversion to blur and preference for clarity^1^ are also shown in literary works and wordings. For instance, in the Chinese fairy tale titled *Pangu Separates the Sky from the Earth*, which introduces the origin of sky and earth, Pangu was born as the first figure in China’s history in the universe with nothing but darkness and chaos. At that time, the sky and the earth were one blurred entity. He felt depressed and outrageous so that he chopped the blurry entity with a hammer, and thus the sky was separated from the earth. This tale may not explain the origin of our world scientifically but is reflective of people’s aversion for chaos and blur to some degree. Similarly, in Dante’s *Divine Comedy*, the rivers in Hell are turbid, while Heaven is a pure and peaceful place. Furthermore, Chinese words, which contain a few characters semantically related to chaos or blur, tend to be negative words, like “
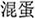
” (‘hundan’ means ‘bastard,’ with the character ‘hun’ meaning ‘chaos’), “
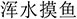
” (‘hunshuimoyu’ means ‘fish in muddied waters,’ with the character ‘hun’ meaning ‘blurry’), whereas those with clear-related characters, like “
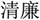
” (‘qinglian’ means ‘incorruptible,’ with the character ‘qing’ meaning ‘clear’), “
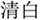
” (‘qingbai’ means ‘innocent,’ with the character ‘qing’ meaning ‘clear’) are regarded as positive words. Taken together, it seems that people tend to view clear objects as good and blurry ones as bad. However, whether there exists a potential association between clarity and affect in human cognition remains unknown.

Abstract concepts (e.g., happiness, status, friendship, morality) are not concrete physically, which means that we cannot perceive or understand them directly through five senses, namely, vision, hearing, taste, touch and smell. Given that they lack the direct references in human’s perception system, then how they are presented and conceptualized in our mind constitutes a foci in cognitive science (Kintsch, 1988; Burgess and Lund, 1997; Glenberg and Kaschak, 2002; Santiago et al., 2011; Casasanto and Bottini, 2014; Winkielman et al., 2018). Accordingly, a number of relevant theories are proposed by linguists and researchers focusing on cognition as well as neuro-science, and an amodal/modal representation debate emerges (Barsalou et al., 2003; Pulvermüller, 2013; Mahon, 2015).

In amodal approaches, concepts are situated in a semantic system (e.g., a feature list, semantic network or frame) separated from the brain’s model systems for perception, action and introspection (Barsalou et al., 2003; Barsalou, 2008). They are represented by abstract modal-free symbols, which redescribe and represent information initially encoded in sensorimotor systems. Accordingly, these accounts have two explicit assumptions: one is that cognitive processes are unconstrained by the structure of the body and brain; the second one is that amodal and non-perceptual symbols underlie the higher-level processes (Winkielman et al., 2015). However, with the advent of embodied cognition, both of them are questioned due to the relatively limited empirical evidence (Barsalou, 1999) on the one hand, and the symbol grounding problem (Harnad, 1990) on the other hand, that is, where do these abstract symbols get meanings from if they are only connected to other meaningless amodal symbols?

Conversely, modal theories, or referred as modality-specific theories, hold abstract concepts are grounded in bodily experiences and emphasize the role of perceptual–conceptual associations in the conceptual process. For instance, the Perceptual Symbol Theory (PST) emphasizes the grounded nature of conceptual representation and the importance of sensorimotor experiences (Barsalou, 1999). According to the PST, modal representations are partially activated during language comprehension, leading to simulation that enables relevant perceptual and motor systems to be activated (Barsalou, 1999, 2008). Moreover, Lakoff and Johnson (1980, 1999) established the Conceptual Metaphor Theory (CMT), proposing that concrete concepts are learned directly through perception, whereas abstract concepts are grounded metaphorically on certain physical dimensions. Specifically, abstract concepts (target domains) are mapped to the certain concrete concepts (source domains), which is determined and facilitated by conceptual metaphors, such as “GOOD IS UP,” “LOVE IS WARMTH,” etc. Similarly, a newer blended view of structure-mapping and embodied cognition has been proposed, with the key role of metaphorical mapping in the path between concrete and abstract concepts emphasized (Marmolejo-Ramos et al., 2017). Another addition to this model is the involvement of contextual elements, that is, abstract concepts are grounded in perceptual experiences within a conversational context and social environment rather than in isolated individuals (Cevasco and Marmolejo Ramos, 2013).

In support of modal theories, behavioral and neural evidence has accumulated. In terms of behavioral findings, both top-down and bottom-up effects are taken as evidence for the perceptual–conceptual associations (for reviews, see Santiago et al., 2011; Winkielman et al., 2015). The top-down effects refer to those that sensorimotor systems are activated during higher-order cognitive processing tasks in which sensorimotor processing is not required necessarily. For example, seeing a certain object activates the general hand shape of grabbing or using it (Klatzky et al., 1989); after reading the word “kick,” the appropriate motor presentation of leg is activated unconsciously (Hauk et al., 2004); participants who scores higher in a measure of chronic loneliness are associated with an increased tendency to take warm baths or showers (Bargh and Shalev, 2012), etc. With respect to the bottom-up effects, a series of studies demonstrate that the performance in those tasks related to conceptual processing may be biased by task-irrelevant perceptual interference. For instance, holding a warm (versus cold) cup of coffee makes people get along with strangers in a more friendly way (Williams and Bargh, 2008); experiencing physical instable conditions can undermine the perceptions of relationship stability (Forest et al., 2015); the perception of size information can interfere the judgment of competition outcomes, i.e., victory or defeat (Yu et al., 2017). Similarly, when participants perceive certain sensorimotor information, regions of the brain related to corresponding conceptual processing are activated and vice versa (Hauk and Tschentscher, 2013; Simmons et al., 2013; Hickok, 2014; Stasenko et al., 2014). Above all, the behavioral and neural studies confirm the perceptual–conceptual associations as well as their significant role in human cognition.

Among the perceptual–conceptual associations, much of this work concerns about affect, which is a term used broadly to encompass emotions, moods and affective valence (Crawford, 2009). It, especially valence, is involved in daily life earlier even when we are children. Kids are able to use expressions as simple as ‘yeahs’ and ‘yucks’ to describe ‘what is good’ and ‘what is bad’ in their mind, with other more complex abstract concepts (e.g., justice, democracy, and value) acquired and understood later in life (Winkielman et al., 2018). It can be seen that affect is a relatively fundamental abstract concept and thus becomes a favored entry point for an abundant body of research on the conceptual process of abstract concepts.

Among them, a great number of studies, under the theoretical framework of embodiment, link it with concrete and physical dimensions, e.g., brightness, spatial position, distance (for reviews, see Meier and Robinson, 2005; Crawford, 2009; Winkielman et al., 2018). For instance, consistent with the metaphor “GOOD IS UP,” positive words are evaluated faster than negative words in the up position while negative words show reaction time advantage in the down position, confirming the perceptual–conceptual association of affect with spatial position (Meier and Robinson, 2004). When experimental stimuli are changed from words to sentences (Marmolejo-Ramos et al., 2014), pictures and faces (Elizabeth Crawford et al., 2006; Mahieu et al., 2014), such metaphor-consistent effect holds true. This effect has also been consolidated in various tasks, involving on-line evaluation (Meier and Robinson, 2004), memory (Crawford et al., 2014), eye-tracking (Gozli et al., 2013), physical movements (Kato et al., 2018), and 3D-space tasks which extend the valence-space metaphor effect to the dimension of 3D space (Marmolejo-Ramos et al., 2018, 2019). Furthermore, the affective perceptual–conceptual associations have been verified when other physical dimensions are involved, including brightness (Meier et al., 2004, 2015; Lakens et al., 2012; Huang et al., 2018), pitch (Weger et al., 2007), taste (Meier et al., 2012), size (Meier et al., 2008), and weight (Zhao et al., 2016).

Given the above-mentioned empirical and theoretical evidence, the perceptual–conceptual associations between affective valence and various physical dimensions seem to be pervasive. Such physical metaphors are suggested to be useful to represent abstract concepts of affective valence (Gibbs, 1992; Glucksberg, 2001). In addition to these physical dimensions, our wordings and literary works have told us the possible association between the physical clarity and abstract valence. However, few empirical studies involve the perception of clarity (with the exception of Yaxley and Zwaan, 2007, demonstrating that during language comprehension, readers mentally stimulate the visibility of objects in terms of visual resolution), which is treated as an important characteristic to describe what we face. On a daily basis, whenever we open our eyes, the clarity serves as the bridge leading us to this world. How about our perception of this world? Is it clear, or is it blurry? How does this clarity interact with our cognition? Evidently, this can be of great significance to our human beings, adding to the conceptual understanding of affective valence by referring to the concrete physical dimensions. Therefore, the present study sought to examine whether clarity is associated with affective valence in cognition.

Here in the present study, altogether five experiments with the Stroop-like paradigm were conducted, which was widely used to detect the perceptual–conceptual association (Meier and Robinson, 2004; Meier et al., 2008; Dudschig and Kaup, 2017; Yu et al., 2017; Huang et al., 2018). With respect to the dimension of clarity, it was manipulated in terms of visual resolution by whether the image including blurred words (low resolution) or not (high resolution), as previously used by McConkie and Loschky (2002) as well as Yaxley and Zwaan (2007). To examine the potential association between the perception of clarity and the conceptualization of affective valence, a totally task-irrelevant manipulation of stimulus visual resolution (Experiments 1, 2, 3) or stimulus valence (Experiments 4, 5) was performed. Experiments 1 through 3 focused on the potential influence of clarity on valence, in which participants were instructed to evaluate words as positive or negative. If the abstract concept of affective valance is associated with the physical perception of clarity, it is expected to observe that participants should show response advantages in the congruent conditions (clear-positive, blurry-negative) than in the incongruent conditions (clear-negative, blurry-positive). As suggested by Lakoff and Johnson (1999), perceptual–conceptual associations are asymmetrical, namely, they argued that abstract concepts are represented in terms of more concrete concepts, but not vice versa (also see Piaget and Inhelder, 1972). Therefore, Experiments 4 and 5 sought to examine the influence of conceptualization on perception, where participants had to categorize the clarity of stimuli as clear or blurry. The hypothesis of asymmetrical association would predict that the response advantages between congruency and incongruency should be absent.

## Experiment 1

Experiment 1 was the first test to examine the clarity–valence association by focusing on the influence of the perception of clarity on the conceptualization of valence.

### Participants

A total of 32 undergraduates and postgraduates (16 females) from Zhejiang University participated in this experiment and were paid RMB¥20 after the whole procedure. They all had normal or correct-to-normal vision. The participants provided their written informed consent before the experiment and all experimental procedures conformed with the Research Ethics Board of Zhejiang University. The study was also reviewed and approved by the Research Ethics Board of Zhejiang University.

### Materials

Sixty positive Chinese words, e.g., “
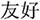
” (‘youhao’ means ‘friendly’), and 60 negative words, e.g., “
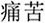
” (‘tongku’ means ‘misery’) were selected as the stimuli (see Appendix Table A). Each word subtended from the participant’s view. We invited 20 undergraduates in Zhejiang University, who didn’t participate in either one of the five experiments, to rate these 120 words in the positive-negative dimension for valence, using a Likert-type scale (1 = extremely negative, 4 = neutral, 7 = extremely positive). The negative words (*M* = 2.42 ms, *SD* = 0.48) were rated as significantly more negative than the positive words (*M* = 5.45 ms, *SD* = 0.44), *t*(118) = 36.33, *p* < 0.001, *d* = 6.69. In terms of the extremity, there was no significant difference between the deviation of the ratings of positive words from the midpoint and that of negative words, *t*(118) = 0.42, *p* = 0.675, *d* = 0.08. The number of stroke was similar for positive and negative Chinese words, *t*(118) = 1.50, *p* = 0.137, *d* = 0.28.

**FIGURE 1:**
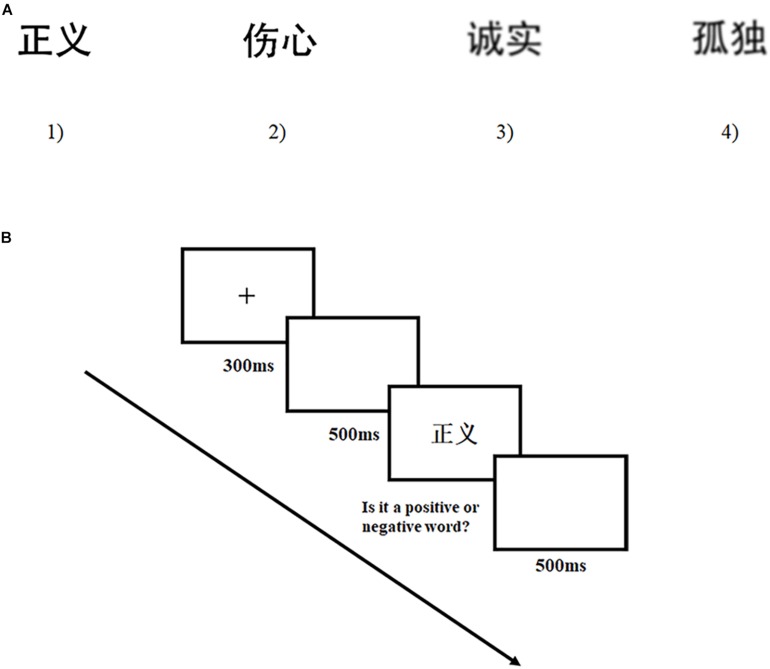
Stimuli and procedure in Experiment 1. **(A)** Four kinds of stimulus used in this experiment. From left to right, the stimuli are (1) clear positive stimulus (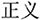, ‘zhengyi’ means ‘justice’); (2) clear negative stimulus (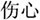, ‘shangxin’ means ‘sad’); (3) blurry positive stimulus (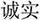, ‘chengshi’ means ‘honest’); (4) blurry negative stimulus (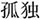, ‘gudu’ means ‘lonely’). **(B)** The sequence of events in one trial (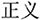, ‘zhengyi’ means ‘justice’).

As for the manipulation of clarity, half of the words were blurred by the *Gaussian Blur* (7 radium), a tool in Photoshop to adjust the resolution of pictures, while the other half maintained clear. Positive and negative words were assigned to be in the clear or blurry condition randomly and presented in a random order. Thus, the factor of clarity cannot function as the cue for the affective valence of displayed words. These words in black (0% gray scale) were displayed on a white background (100% gray scale, see Figure 1A).

### Procedure and Design

In the whole procedure, participants sat in front of a 14-inch computer screen and maintained a distance of 60 cm. Every trial began with a red fixation “+” presented for 300 ms at the center of the screen. Then, a blank interface appeared for 500 ms. After that, a target word was presented and participants were asked to evaluate it as positive or negative as quickly and accurately as possible. Responses were made by pressing one of the two target keys (“P” key for positive words, “Q” key for negative words). The next trial would begin after 500 ms (see Figure 1B). This experiment consisted of 120 trials, 20 for practice. After the practice section, there were two blocks with each one having 50 trials and participants had a chance to take a 2-min rest between the two blocks. A post-experiment verbal report was conducted in each experiment, indicating that participants did not figure out the true purposes of these experiments.

Here were two measures for performance: reaction time (RT) and accuracy rate (AR). In cognitive sciences, RT and AR are two important indicators to quantify the performance, but there may be contradictory or inconsistent results in these two aspects and occasional effects of speed-accuracy trade-off. Therefore, as suggested before (Hughes et al., 2014; Draheim et al., 2016; Vandierendonck, 2017), we combined speed and accuracy into a unified measure, inverse efficiency score (IES), to determine the relation between the perception of clarity and the conception of valence (RT and AR results in each experiment are also presented in Appendix Table B). IES is the oldest and the most frequently used measure integrating RT and AR (Vandierendonck, 2017) and has been applied by a line of empirical work (Kunar et al., 2007; Kerzel, 2019; Kerzel and Witzel, 2019; Machlin et al., 2019; Ondish et al., 2019). According to Townsend and Ashby (1978), the IES is calculated by dividing the average correct reaction time with the proportion of correct answers, that is, IES = RT/AR. For example, if someone responded correctly in every single trial, then the IES value would be equal to RT, whereas someone who got 90% correct answers would have a score of RT divided by 0.90. Similar to RTs, the higher IES values indicate poorer performance.

### Data Analyses

In this experiment and the other four experiments below, the dependent variable was IES value with the valence (positive vs. negative) and clarity (clear vs. blurry) of stimuli as the independent variables. Thus, standard repeated two-way analyses of variance (ANOVA) were employed in each experiment. In line with the notion of multiverse analyses (Steegen et al., 2016), the supplementary analyses were performed via permutation tests for linear models (see Supplementary Material). Different from commonly used parametric tests, where dataset are regarded as a sample from a normal or well-known distribution, permutation methods are based on randomization, with the idea of generating reference distribution through the recalculation of a statistic for many permutations of the data (Ernst, 2004). Permutation tests have shown edges in the context of RT data (Morís Fernández and Vadillo, 2019).

### Results and Discussion

After excluding the outliers in which the RTs deviated more than 2.439 SD from the mean, data of 3,107 trials were analyzed (exclusion rate is 2.9%). This SD cut-off is an adjusted one according to sample size for outlier elimination. The adjustment of SD cut-off was proposed to minimalize the estimation bias (see Table 4 inVan Selst and Jolicoeur, 1994; Cousineau and Chartier, 2010; Marmolejo-Ramos et al., 2015), and has been employed in some empirical work on RT, though rare (e.g., Chen et al., 2018). Even the 3 SD criteria was used, as previously suggested in the similar studies, the consistent results were found on IES.

**FIGURE 2:**
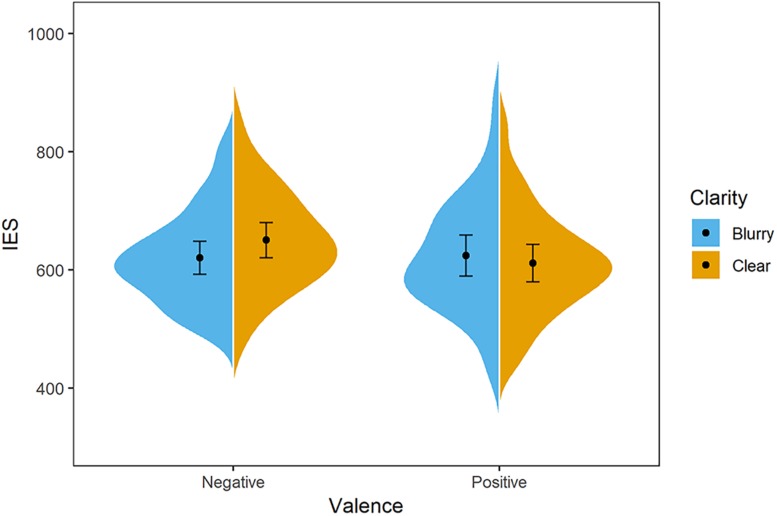
Distribution of participants’ IES in Experiment 1. The blue/orange areas represent the IES’s probability density, with the black point representing mean and error bars indicating the standard error (±SE). Negative-blurry condition: *M* = 620 ms, *SE* = 14; negative-clear condition: *M* = 650 ms, *SE* = 15; positive-blurry condition: *M* = 624 ms, *SE* = 17; positive-clear condition: *M* = 611 ms, *SE* = 16.

A two-way (valence: positive vs. negative; clarity: clear vs. blurry) repeated analysis of variance (ANOVA) was conducted on IES value. The main effect of valence was significant, *F*(1,31) = 5.81, *p* = 0.022, $\eta_{p}^{2}$ = 0.16. Specifically, the positive words (*M* = 617 ms, *SD* = 90) were evaluated with a better performance than the negative words (*M* = 635 ms, *SD* = 75). The main effect of clarity was not significant, *F*(1,31) = 2.40, *p* = 0.131, $\eta_{p}^{2}$ = 0.07. Furthermore, the interaction effect between clarity and valence was significant, *F*(1,31) = 8.15, *p* = 0.008, $\eta_{p}^{2}$ = 0.21. The simple effect analysis showed that negative words were evaluated better in the blurry condition (*M* = 620 ms, *SD* = 78) compared to the clear condition (*M* = 650 ms, *SD* = 83), *t*(31) = 2.90, *p* = 0.007, *d* = 1.03, whereas negative words (*M* = 650 ms, *SD* = 83) were evaluated more poorly relative to positive words (*M* = 611 ms, *SD* = 88) in the clear condition, *t*(31) = 3.51, *p* = 0.001, *d* = 1.24 (see Figure 2).

Here, a clarity–valence congruency effect is demonstrated, i.e., positive and negative words are evaluated better in the clarity–valence congruent condition (clear-positive, blurry-negative). Such findings support that the perception of clarity and the concept of affect is associated.

## Experiment 2

Experiment 1 provided the initial evidence to support the association between clarity and valence. However, given that the clear stimuli were brighter than the blurry stimuli due to the manipulation of visual resolution achieved by the *Gaussian Blur*, the clarity–valence congruency effect might result from the influence of brightness rather than clarity, as the brightness–valence association also biases the performance of participants in the similar evaluation tasks (Meier et al., 2004, 2015; Lakens et al., 2012). To rule out the possible explanation of brightness, Experiment 2 with an anti-color design was conducted. In this setting, the clear and blurry stimuli maintained the same visual-resolution level with Experiment 1, but showed in the opposite brightness level with Experiment 1. If the results of Experiment 1 were due to the brightness–valence association, the effect observed in Experiment 1 would be absent; otherwise, the identical effect in this experiment would be detected.

### Participants and Design

Another 32 undergraduates and postgraduates (18 females) participated in this experiment. The participants provided their written informed consent before the experiment and all experimental procedures conformed with the Research Ethics Board of Zhejiang University. The study was also reviewed and approved by the Research Ethics Board of Zhejiang University.

Contrary to Experiment 1 where words in black (0% gray scale) were presented on a white (100% gray scale) background, the colors of words and the background in Experiment 2 were reversed so that the blurry stimuli were brighter than the clear stimuli in the dimension of illumination. Except for this, other aspects of the manipulation and procedure here were identical to those in Experiment 1.

### Results and Discussion

**FIGURE 3:**
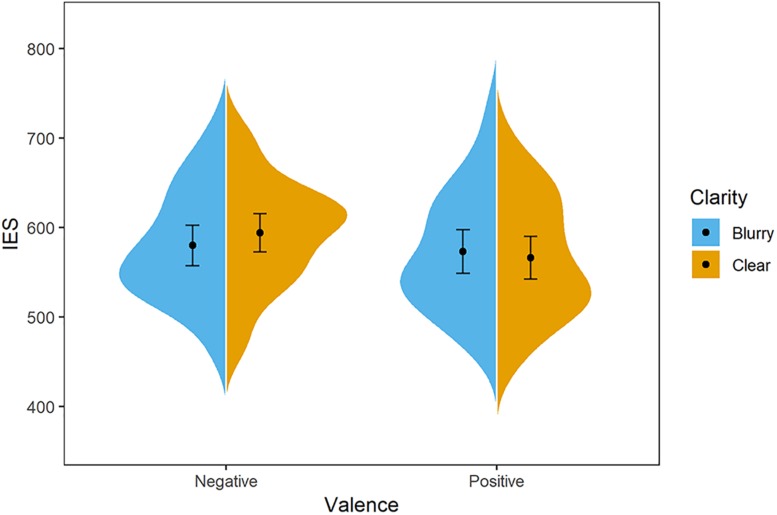
Distribution of participants’ IES in Experiment 2. The blue/orange areas represent the IES’s probability density, with the black point representing mean and error bars indicating the standard error (±SE). Negative-blurry condition: *M* = 580 ms, *SE* = 11; negative-clear condition: *M* = 594 ms, *SE* = 11; positive-blurry condition: *M* = 573 ms, *SE* = 12; positive-clear condition: *M* = 566 ms, *SE* = 12.

After excluding the outliers according to the same criteria as Experiment 1, data of 3,102 trials were analyzed (exclusion rate is 3.1%). For the IES value, the main effect of clarity was not significant, *F*(1,31) = 1.71, *p* = 0.442, $\eta_{p}^{2}$ = 0.02. But the main effect of valence was significant, *F*(1,31) = 5.25, *p* = 0.029, $\eta_{p}^{2}$ = 0.15, and the positive words (*M* = 570 ms, *SD* = 66) were evaluated with a better performance than the negative words (*M* = 587 ms, *SD* = 57). The interaction effect between clarity and valence was significant, *F*(1,31) = 6.90, *p* = 0.013, $\eta_{p}^{2}$ = 0.18. The simple effect analysis showed that negative words were evaluated marginally better in the blurry condition (*M* = 580 ms, *SD* = 63) compared to the clear condition (*M* = 594 ms, *SD* = 60), *t*(31) = 1.90, *p* = 0.067, *d* = −0.67, whereas negative words (*M* = 594 ms, *SD* = 60) were evaluated more poorly relative to positive words (*M* = 566 ms, *SD* = 66) in the clear condition, *t*(31) = 3.50, *p* = 0.001, *d* = 1.24 (see Figure 3).

The results confirm the clarity–valence congruency effect in the anti-color condition, where the blurry stimuli were brighter than the clear stimuli. Thus, the effect in both experiments is proved to be the result of clarity–valence association rather than the influence of brightness.

## Experiment 3

Lakoff (1993, 2012) suggested that the metaphorical conceptual representation functions unconsciously and automatically. With the co-occurrence of task-relevant and task-irrelevant stimuli, the Stroop-like paradigm can meet the need to explore the automatic nature of perceptual–conceptual associations, which partly accounts for why it is favored by this relevant body of studies (Meier and Robinson, 2004; Meier et al., 2004, 2015; Gliksman et al., 2016). In addition to the Stroop-like paradigm, a response-deadline procedure, in which participants had to respond faster, was employed in Experiment 3 to better support the automaticity of clarity–valence interaction. This procedure is widely employed to examine the naturality and automaticity of relevant processes on the one hand (Draine and Greenwald, 1998; Payne, 2001; Meier et al., 2004; Meier et al., 2008), and on the other hand offers access to detecting the earliest and most unconscious stages of stimuli evaluation (Meier et al., 2008).

### Participants

Participants were 32 undergraduates and postgraduates (19 females) from Zhejiang University. They provided their written informed consent before the experiment and all experimental procedures conformed with the Research Ethics Board of Zhejiang University. The study was also reviewed and approved by the Research Ethics Board of Zhejiang University.

### Materials and Procedure

The manipulation of stimuli and almost all aspects of procedure in Experiment 3 were the same with Experiment 1, except for the response-deadline design. Specifically, when stimuli were displayed, participants were instructed to respond within a time limit. A 700-ms response window was used, similar to other similar researches (Robinson et al., 2005; Meier et al., 2008). If participants responded slowly, i.e., exceeding 700 ms, they would be informed by a notice “
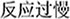
” (‘fanyingguoman’ means ‘too slow’) on the center of the screen. Given that the response-deadline design may cause the low-accuracy trials, every word was evaluated twice to obtain enough valid trials for analysis, so total 200 trials were in this experiment. Another 20 trials were included for practice. After the practice section, there were four blocks with each one having 50 trials and participants had a chance to take a 2-min rest between each two blocks.

### Results and Discussion

With the deadline-response procedure, participants in Experiment 3 (*M* = 533 ms, *SD* = 28) responded significantly faster than those in Experiment 1 (*M* = 613 ms, *SD* = 82), *t*(62) = 5.23, *p* < 0.001, *d* = 1.34. It was indicated that the response-deadline procedure forced participants to speed up, facilitating the exploration of unconscious and automatic cognitive processes.

**FIGURE 4:**
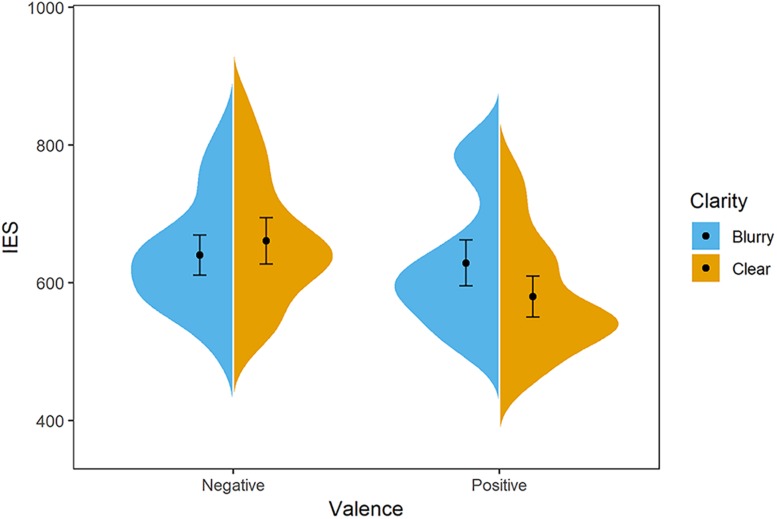
Distribution of participants’ IES in Experiment 3. The blue/orange areas represent the IES’s probability density, with the black point representing mean and error bars indicating the standard error (±SE). Negative-blurry condition: *M* = 640 ms, *SE* = 14; negative-clear condition: *M* = 661 ms, *SE* = 16; positive-blurry condition: *M* = 629 ms, *SE* = 16; positive-clear condition: *M* = 580 ms, *SE* = 15.

In this more challenging task, two participants’ data (two females) were removed due to low accuracy (lower than 50%). To achieve a balanced sample size in each experiment, we recruited another two female participants to be involved in Experiment 3. The ANOVA analysis on IES showed that both the main effect of valence [*F*(1,31) = 18.47, *p* < 0.001, $\eta_{p}^{2}$ = 0.37] and clarity [*F*(1,31) = 5.01, *p* = 0.032, $\eta_{p}^{2}$ = 0.14] were significant. On the one hand, the positive words (*M* = 604 ms, *SD* = 82) were evaluated with a better performance than the negative words (*M* = 650 ms, *SD* = 78). On the other hand, participants performed better in the clear condition (*M* = 620 ms, *SD* = 77) than in the blurry condition (*M* = 634 ms, *SD* = 75). The interaction effect between clarity and valence was significant, *F*(1,31) = 10.28, *p* = 0.003, $\eta_{p}^{2}$ = 0.25. The simple effect analysis showed that positive words (*M* = 580 ms, *SD* = 83) were evaluated better than negative words (*M* = 661 ms, *SD* = 93) in the clear condition, *t*(31) = 5.47, *p* < 0.001, *d* = 1.96. As for the positive words, participants showed IES advantage in the clear condition (*M* = 580 ms, *SD* = 83) relative to the blurry condition (*M* = 630 ms, *SD* = 93), *t*(31) = 4.34, *p* < 0.001, *d* = 1.56 (see Figure 4). The results indicate that the clarity–valence congruency effect still exists when participants are under time pressure.

With the response-deadline procedure employed in this experiment, we conclude that the perception of clarity has an effect on the conceptualization of valence in an automatic way, which is consistent with the ideas of Lakoff (1993, 2012). However, it is needed to note that the concept of automaticity is still controversial and measured differently in terms of one or various features (Bargh, 1994; Moors and De Houwer, 2006). According to Moors and De Houwer (2006), the construct of automaticity encompasses four types of features, namely, goal-related features (e.g., unintentional, uncontrolled, purely stimulus driven), unconscious, efficient and fast, and it is better to investigate them separately. However, it is less practical to consider all dimensions of automaticity and few processes may be automatic in relations to all different criteria (e.g., Bargh, 1994). In this regard, *fast* is selected here due to that: (1) the diagnosis and manipulation of it are of high operability through a time-pressured design; (2) it is closely linked with and can be indicative of other features (Moors and De Houwer, 2006). Along with the Stroop-like paradigm which contributes to the exploration of the unintentional feature of automaticity, the findings here can, at least, extend the results of Experiments 1 and 2 toward the automatic nature of clarity–valence association.

## Experiment 4

Experiments 1 through 3 confirmed the clarity–valence association in the concrete-to-abstract direction, i.e., the perception of clarity biases the evaluation of affective valence. While, it is not clear whether such association can be present in the reverse abstract-to-concrete direction. To explore this question, in Experiment 4, we focused on the potential influence of the conceptualization of valence on the perception of clarity. Different from previous experiments, participants were asked to evaluate the clarity information of stimuli in this experiment.

### Participants

Thirty-two undergraduates and postgraduates (17 females) from Zhejiang University participated in this experiment. The participants provided their written informed consent before the experiment and all experimental procedures conformed with the Research Ethics Board of Zhejiang University. The study was also reviewed and approved by the Research Ethics Board of Zhejiang University.

### Materials and Procedure

Most aspects here were the same as those in Experiment 1 except that participants were asked to categorize every word presented as clear or blurry. Responses were made by pressing one of the two target keys (“K” for clear words, “S” for blurry words).

### Results and Discussion

**FIGURE 5:**
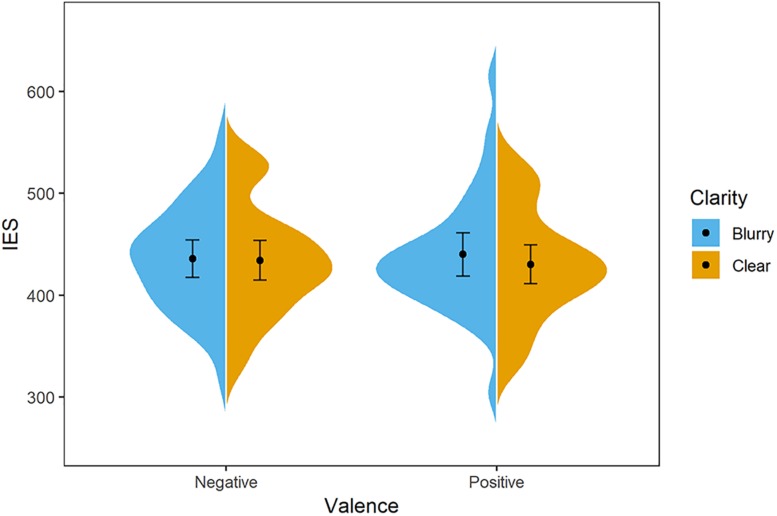
Distribution of participants’ IES in Experiment 4. The blue/orange areas represent the IES’s probability density, with the black point representing mean and error bars indicating the standard error (±SE). Negative-blurry condition: *M* = 436 ms, *SE* = 9; negative-clear condition: *M* = 434 ms, *SE* = 9.5; positive-blurry condition: *M* = 440 ms, *SE* = 10; positive-clear condition: *M* = 430 ms, *SE* = 9.

After excluding outliers, data of 3,118 trials were analyzed (exclusion rate is 2.6%). The ANOVA analyses on IES value showed that neither the main effect of valence [*F*(1,31) < 0.01, *p* = 0.959, $\eta_{p}^{2}$ < 0.01], the main effect of clarity [*F*(1,31) = 1.22, *p* = 0.278, $\eta_{p}^{2}$ = 0.04], nor the interaction effect between clarity and valence interaction [*F*(1,31) = 1.04, *p* = 0.316, $\eta_{p}^{2}$ = 0.03] was significant (see Figure 5). The results above indicate that the clarity–valence congruency effect disappears in the clarity-evaluation task.

## Experiment 5

Contrary to Experiment 1 to Experiment 3, the null effect of clarity–valence congruency was found in the clarity-evaluation task of Experiment 4. One possibility to explain this inconsistency is that the valence information of the stimuli was completely ignored in Experiment 4 given that participants unnecessarily processed word meaning to complete the clarity-evaluation task. To further examine whether the clarity–valence congruency effect in the abstract-to-concrete dimension exists, the activation of valence information should be boosted. We simultaneously adopted both the valence-evaluation and the clarity-evaluation task in Experiment 5, which was inspired by the relevant research (Shen et al., 2016; Huang et al., 2018). Specifically, participants were instructed to evaluate either the valence (positive vs. negative) or the clarity (clear vs. blurry) information of words displayed in each trial according to the task cue displayed randomly.

### Participants

Participants were 32 undergraduates and postgraduates (18 females) from Zhejiang University. They provided their written informed consent before the experiment and all experimental procedures conformed with the Research Ethics Board of Zhejiang University. The study was also reviewed and approved by the Research Ethics Board of Zhejiang University.

### Materials and Procedure

A total of 112 words (56 positive words and 56 negative words) used in this experiment were randomly selected from the 120-word pool in Experiment 1. Here, participants engaged in a mixed task, in which they were instructed to evaluate either the valence or the clarity information of target words. The different task (valence-evaluation or clarity-evaluation) was presented randomly in each trial.

Similar with the design of Shen et al. (2016), the experiment began with a red fixation “+” presented for 300 ms at the center of the screen. The following interface was a blank one. After that, a task cue (i.e., 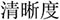, ‘qingxidu’ meaning ‘clarity’ or 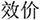, ‘xiaojia’ meaning ‘valence’) was presented for 1,500 ms, informing participants to evaluate the following stimulus as positive/negative or clear/blurry. Then, a word was presented for participants to respond. When participants saw “
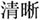
” (‘qingxidu’ means ‘clarity’), they would evaluate words as clear or blurry. Similarly, if they saw “
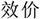
” (‘xiaojia’ means ‘valence’), they needed to evaluate words as positive or negative. In the valence-evaluation task, participants pressed “P” key (for positive word) or “Q” key (for negative word) key. Whereas the key-pressing mode in the clarity-evaluation task was counterbalanced between participants. Specifically, half of the participants pressed the “S” key for clear words and the “K” key for blurry words while in reserve for the other half. This manipulation was to rule out the potential interference of the layout of keys in the keyboard. The next trial began after a blank interface for 500 ms.

In Experiment 5, there were four combinations (2 positive/negative × 2 clear/blurry). The experiment included 112 trials in total: 16 trials for practice and 24 trials for each combination condition. After the practice section, there were two blocks with each one having 96 trials and participants had a chance to take a 2-min rest between the two blocks. Other aspects were the same as those in Experiment 1.

### Results and Discussion

**FIGURE 6:**
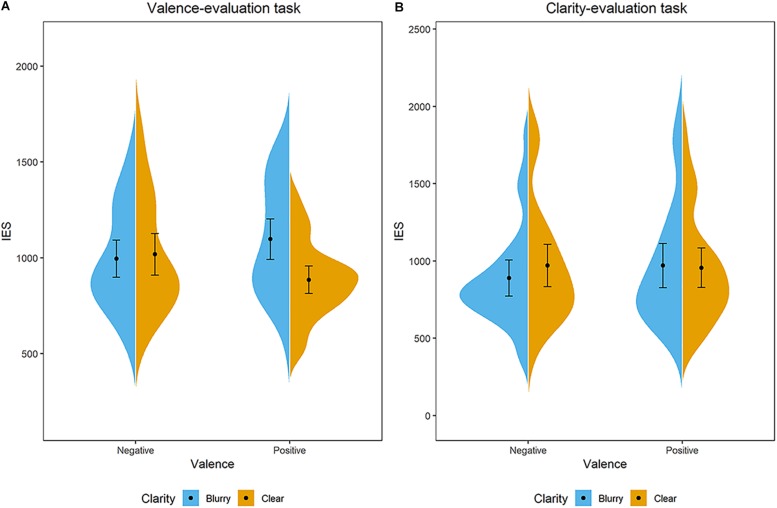
Distribution of participants’ IES in the **(A)** valence-evaluation task and **(B)** clarity-evaluation task of Experiment 5. The blue/orange areas represent the IES’s probability density, with the black point representing mean and error bars indicating the standard error (±SE). For the valence-evaluation task, negative-blurry condition: *M* = 996 ms, *SE* = 48; negative-clear condition: *M* = 1,019 ms, *SE* = 53; positive-blurry condition: *M* = 1,098 ms, *SE* = 52; positive-clear condition: *M* = 886 ms, *SE* = 35. For the clarity-evaluation task, negative-blurry condition: *M* = 890 ms, *SE* = 57; negative-clear condition: *M* = 971 ms, *SE* = 68; positive-blurry condition: *M* = 970 ms, *SE* = 70; positive-clear condition: *M* = 956 ms, *SE* = 63.

After excluding outliers, data of 1,434 trials in the valence-evaluation task were analyzed (exclusion rate is 6.6%). According to the ANOVA results, the main effect of valence was insignificant, *F*(1,31) = 0.47, *p* = 0.498, $\eta_{p}^{2}$ = 0.04, but the main effect of clarity was significant, *F*(1,31) = 21.07, *p* < 0.001, $\eta_{p}^{2}$ = 0.41, with stimuli evaluated better in the clear condition (*M* = 952 ms, *SD* = 236) relative to in the blurry condition (*M* = 1,047 ms, *SD* = 261). Furthermore, the clarity × valence interaction was significant, *F*(1,31) = 18.55, *p* < 0.001, $\eta_{p}^{2}$ = 0.37. Specifically, negative words (*M* = 996 ms, *SD* = 269) were evaluated better than positive words (*M* = 1,098 ms, *SD* = 294) in the blurry condition, *t*(31) = 2.77, *p* = 0.009, *d* = 1.00; positive words (*M* = 886 ms, *SD* = 198) were evaluated better than negative words (*M* = 1,019 ms, *SD* = 301) in the clear condition, *t*(31) = 3.91, *p* < 0.001, *d* = 1.40. As for the positive words, participants showed IES advantage in the clear condition (*M* = 886 ms, *SD* = 198) relative to the blurry condition (*M* = 1,098 ms, *SD* = 294), *t*(31) = 5.24, *p* < 0.001, *d* = 1.88 (see Figure 6A).

In the clarity-evaluation task, data of 1,491 trials were analyzed after excluding outliers (exclusion rate is 2.9%). It was demonstrated from the ANOVA analysis in terms of IES that neither the main effect of valence [*F*(1,31) = 0.95, *p* = 0.338, $\eta_{p}^{2}$ = 0.03], the main effect of clarity [*F*(1,31) = 1.20, *p* = 0.281, $\eta_{p}^{2}$ = 0.04], nor the interaction effect between clarity and valence interaction [*F*(1,31) = 3.00, *p* = 0.093, $\eta_{p}^{2}$ = 0.09] was significant (see Figure 6B).

In this experiment, the significant clarity × valence interaction in the valence-evaluation task suggested that participants conducted valence information processing, meanwhile confirming the congruency effect in the valence-evaluation task again. Even if so, no clarity × valence interaction was shown in the clarity-evaluation task, indicating that the conceptualization of affective valence does not modulate the perception of clarity.

## General Discussion

In the present study, five experiments were conducted to examine the potential perceptual–conceptual association of affective valence with clarity. In Experiment 1, a valence-evaluation task with an irrelevant manipulation of clarity was employed, in which evaluation showed a performance (i.e., IES) advantage in the congruent conditions (clear-positive, blurry-negative). This effect can be called as the clarity–valence congruency effect, and the probable influence of brightness was ruled out (Experiment 2). Such effect was also verified in the deadline-response procedure (Experiment 3), suggesting the automatic nature of clarity–valence interaction. Experiments through 1 to 3 confirmed the influence of clarity perception on the conceptualization of valence, while Experiments 4 and 5 focused on the other direction around this association. The findings showed that such clarity–valence congruency effect was absent in the clarity-evaluation task (Experiment 4), even when participants were forced to process the valence information of stimuli (Experiment 5), confirming the unidirectional association between clarity and valence.

### Clarity–Valence Congruency Effect

Here in each valence-evaluation task, the interaction effect between clarity and valence was significant. Specifically, positive words showed evaluation advantage in the clear condition whereas negative words showed evaluation advantage in the blurry condition, demonstrating the clarity–valence congruency effect. The current findings are consistent with the pioneering study on the space–valence association (Meier and Robinson, 2004), as well as a line of subsequent studies concerning the perceptual–conceptual associations with various physical dimensions (Marmolejo-Ramos et al., 2014; Meier et al., 2015; Damjanovic and Santiago, 2016; Zhao et al., 2016; Castaño et al., 2018; Chen et al., 2018; Woodin and Winter, 2018). For example, Zhao et al. (2016) examined the metaphoric link between weight perception and emotional words through a priming paradigm, another dominant paradigm used in this body of research. According to their results, the weight perception biased the judgments of emotional words, indicating a congruency effect (light-positive, heavy-negative).

However, such congruency effect is not always the case. Crawford et al. (2014) found a memory advantage for words that had been studied in locations that were incongruent with GOOD IS UP conceptual metaphor, i.e., participants showed facilitated performance in a memory-related task when negative words were presented in higher positions relative to lower positions.

As mentioned at the very beginning of this paper, the link between clarity and affective valence may have bases in our daily life, e.g., literary works as well as linguistic expressions. Here in this study, such association is activated in each valence-evaluation task with an irrelevant manipulation of clarity, suggesting that clarity–valence association is rooted in our cognitive system. Similar with other physical dimensions linked with affect, clarity also serves as an embodied grounding for us to reason and comprehend the abstract concept—affect. The clarity–valence congruency effect confirmed in the present study extends the affective perceptual–conceptual association to the dimension of clarity.

To our knowledge, this is the first effort in this line focusing on clarity–valence association. Along with other congruency effects, the clarity–valence congruency effect found in the present study enriches the embodied view of cognition, including PST (Barsalou, 1999, 2008; Barsalou et al., 2003) and CET (Lakoff and Johnson, 1980, 1999), from a new dimension on the one hand. On the other hand, the present findings provide important implications for the evaluative judgments in daily life. The reason why we prefer HD screens and dislike the blurry view on smoggy days is normally regarded as our preference for more visual details. Here in our research, the findings suggest that these phenomena may partly be the consequences of an automatic tendency to view blurrier objects as worse.

### Unidirectionality of the Clarity–Valence Association

The current study suggests that the clarity–valence association is unidirectional, i.e., the perception of clarity affects the affective conceptual processes, whereas the processing of valence information does not have an effect upon the perception in terms of clarity. This is consistent with a line of studies which only indicated the perceptual–conceptual associations in the concrete-to-abstract direction but not vice versa. For example, Boot and Pecher (2010) investigated the metaphorical mapping for SIMILARITY IS CLOSENESS, finding out that the similarity judgment (abstract dimension) was biased by the manipulation of distance, while distance judgment was not affected by similarity. The similar unidirectionality was also reported in space–time (Boroditsky, 2000; Casasanto and Boroditsky, 2008), verticality–brightness (Meier et al., 2004), as well as weight–valence association (Zhao et al., 2016).

These empirical evidence are in line with the ideas of CET (Lakoff and Johnson, 1999), which postulates that the metaphorical associations will only be activated in the concrete-to-abstract direction because we borrow concrete concepts to enable the conceptualization of abstract concepts but not vice versa. Similarly, Piaget and Inhelder (1972) posited that abstract concepts are scaffolded onto concrete concepts developed earlier in life through perceptual and motor experiences.

On contrary, PST (Barsalou, 1999, 2008) favors the bidirectional view, that is, conceptual and perceptual processes are interacted because their representations share the same resources. Furthermore, Lee and Schwarz (2012) argued that the correlation of conceptual representations with perceptual experiences underlie the mechanism of metaphorical associations, and thus abstract and concrete concepts tend to interact with each other. Accordingly, an increasing number of related research have accumulated in recent years, involving the association between physical and interpersonal warmth (Williams and Bargh, 2008), temperature and loneliness (Zhong and Leonardelli, 2008), size and competition outcome (Yu et al., 2017), as well as physical and conceptual magnitude (Gliksman et al., 2016).

The reason behind the mixed results concerning the directionality of perceptual–conceptual associations remains uncertain yet but becomes a new foci of researchers. In support of bidirectional view, Huang et al. (2018) argued that the unidirectionality verified in experiments with Stroop-like paradigm (e.g., Meier et al., 2004) may be the result of low-level activation of abstract information. With the activation of valence information boosted, they confirmed the bidirectionality of brightness–valence association. Given that the clarity–valence congruency effect in the abstract-to-concrete direction was still absent even when the activation of valance information was boosted, this account is not valid enough to account for the unidirectionality found in this study.

Furthermore, Santiago et al. (2012) held that directionality may depend on language usage frequency. They illustrated that if language usage shows an asymmetric pattern (e.g., people talk about time in terms of space much more often than space in terms of time), associations would be unidirectional. In contrast, with a symmetric pattern (e.g., talking about number in terms of size as often as talking about size in terms of number), bidirectionality would be demonstrated. According to this account, the clarity–valence association is likely to be unidirectional because we often borrow clarity information to express positive or negative meanings [e.g., “
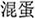
” (‘hundan’ means ‘bastard’) is a negative word, “
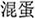
” (‘qinglian’ means ‘incorruptible’) is a positive word], whereas we hardly talk about clarity through valence information. On the other hand, the absence of clarity–valence congruency effect in the abstract-to-concrete may be due to that affect is grounded in many physical dimensions, like space, size, and pitch (Zhao et al., 2016). In this regard, clarity is only one of many concrete sources for the grounding of affective valence, and hence the conceptualization of valence information does not affect the perception related to clarity. We assume this account makes sense and provides a reasonable explanation for the unidirectionality of the clarity–valence association. But more empirical evidence and theoretical models are needed for the directionality-related issues.

## Conclusion

The results of the present study demonstrate the clarity–valence congruency effect, i.e., words show evaluation advantages in the congruent conditions (clear-positive, blurry-negative), suggesting the automatic perceptual–conceptual association between clarity and affective valence. Besides, the conceptualization of valence does not affect the perception of clarity in the clarity-evaluation task, indicating that the clarity–valence association is unidirectional. The current study, to our knowledge, is the first behavioral effort in extending the affective perceptual–conceptual associations to the dimension of clarity, proposing and confirming the clarity–valence congruency effect. To anticipate, future research can be extended to the mechanism underlying this congruency effect and also some probable neurological evidences in this line.

## Data Availability

The raw data supporting the conclusions of this manuscript will be made available by the authors, without undue reservation, to any qualified researcher.

## Ethics Statement

The participants provided their written informed consent before the experiment and all experimental procedures conformed with the Research Ethics Board of the Zhejiang University.

## Author Contributions

JL and YL conceived and designed the experiments. YL, JY, and JL performed the experiments, collected the data, and performed the data analyses. All authors contributed to the interpretation of results and the writing of the manuscript and approved the final version of the manuscript for submission.

## Conflict of Interest Statement

The authors declare that the research was conducted in the absence of any commercial or financial relationships that could be construed as a potential conflict of interest.

## Footnotes

1 This term refers to the quality of being clear to see in the present study. At the operational aspect, visual resolution, a quantifiable index, is usually used as the indicator of visual clarity (McConkie and Loschky, 2002). In this paper, we use the term “clarity” mostly, but use “visual resolution” when introducing the manipulation of clarity.

## Appendix

**TABLE A:** Chinese Affective Words Used in the Present Study With English Translation.

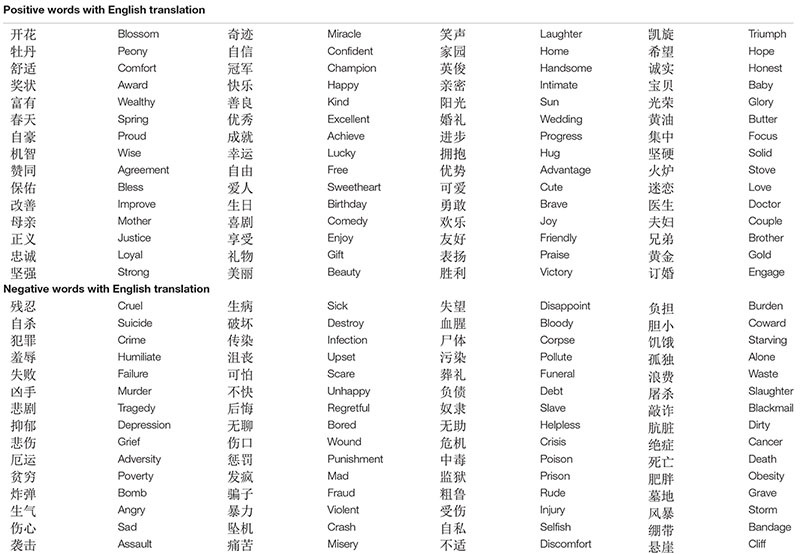

**TABLE B:** Mean Reaction Time and Accuracy Rate in Each Experiment.

**Mean**								
**Experiment**	**Negative-blurry**		**Negative-clear**		**Positive-blurry**		**Positive-clear**	
	**RT (ms)**	**AR**	**RT (ms)**	**AR**	**RT (ms)**	**AR**	**RT (ms)**	**AR**
1 (*n* = 32)	612	0.99	627	0.96	609	0.98	603	0.99
	(81)	(0.02)	(86)	(0.04)	(92)	(0.04)	(91)	(0.02)
2 (*n* = 32)	562	0.97	562	0.95	553	0.97	553	0.98
	(59)	(0.05)	(56)	(0.06)	(64)	(0.04)	(64)	(0.03)
3 (*n* = 32)	541	0.89	539	0.85	531	0.87	523	0.93
	(30)	(0.09)	(30)	(0.14)	(31)	(0.13)	(38)	(0.07)
4 (*n* = 32)	431	0.99	429	0.99	431	0.98	424	0.99
	(51)	(0.02)	(53)	(0.03)	(57)	(0.03)	(53)	(0.02)
5-V (*n* = 32)	964	0.97	977	0.95	1,030	0.94	882	0.99
	(263)	(0.05)	(309)	(0.05)	(285)	(0.09)	(196)	(0.02)
5-C (*n* = 32)	839	0.95	916	0.95	888	0.93	847	0.89
	(282)	(0.09)	(334)	(0.08)	(326)	(0.08)	(329)	(0.09)

*Standard deviations are in parentheses. RT, reaction time; AR, accuracy rate; 5-V, valence-evaluation task in Experiment 5; 5-C, clarity-evaluation task in Experiment 5.*
