# Supplementary material for: Why Smoggy Days Suppress Our Mood: Automatic Association Between Clarity and Valence
Source: Front Psychol. 2019 Jul 10;10:1580. doi: 10.3389/fpsyg.2019.01580 (PMC6635603; doi:10.3389/fpsyg.2019.01580)
Supplement: Supplementary file 1 [file Data_Sheet_1.pdf]

Supplementary material

The same raw data in this study were analysed via permutation-based tests for linear models. Specifically, the function of "aovp" in the R package "lmPerm" was used for the permutation-based two-way repeated ANOVA (Kabacoff, 2011). Different from the F-test normally used in ANOVA, the *p*-value in the permutation test was calculated based on randomization by recalculating a statistic for many permutations of the data (Ernst, 2004), rather than F-distribution. Furthermore, it is no need to preprocess dataset for outlier elimination or transformation (Morís Fernández and Vadillo, 2019). To note, all correct responses were analysed and the permutation procedure here was repeated 5000 times. The results showed in Table 1 indicate that the interaction effect between valence and clarity in each valence-evaluation task was significant, including Experiment 1 ($p < .001$), Experiment 2 ($p = .024$), Experiment 3 ($p < .001$), and the valence-evaluation task in Experiment 5 ($p < .001$), whereas the interaction effect was insignificant in Experiment 4 ($p = 1$) and the clarity-evaluation task in Experiment 5 ($p = .186$).

TABLE 1 | The permutation-based ANOVA results of IES in Experiment 1 through 5

**Experiment**	**Main effect of valence**		**Main effect of clarity**		**Interaction effect**	
	**Iter**	***p***	**Iter**	***p***	**Iter**	***p***
Exp 1	5000	< .001***	51	1	5000	< .001***
Exp 2	5000	< .001***	51	1	4140	.024*
Exp 3	5000	< .001***	5000	< .001***	5000	< .001***
Exp 4	51	1	5000	.002**	51	1
Exp 5_val	2304	.042*	5000	< .001***	5000	< .001***
Exp 5_cla	1589	.060	1882	.051	441	.186

Note. Exp 5_val indicates the valence-evaluation task in Experiment 5; Exp 5_cla indicates the clarity-evaluation task in Experiment 5. *p*-value: 0 '***' .001 '**' .01 '*' .05.

To further examine the interaction effects, the pairwise comparison between the congruent and the incongruent condition (i.e., Negative-Blurry vs. Negative-Clear; Positive-Clear vs. Positive-Blurry) was implemented via the function of "wilcoxsign_test" in the R package "coin", which fits the repeated measures (Kabacoff, 2011).

In Experiment 1, negative words were evaluated better in the blurry condition (M = 647 ms, SD = 216) compared to the clear condition (M = 673 ms, SD = 236), z = 2.74,

$p = .006$, whereas positive words tended to be evaluated better in the clear condition ($M = 626$ ms, $SD = 198$) than in the blurry condition ($M = 645$ ms, $SD = 240$), though insignificant, $z = 1.46$, $p = .142$. But another pairwise comparison performed by us demonstrated that negative words ($M = 673$ ms, $SD = 236$) were evaluated more poorly relative to positive words ($M = 626$ ms, $SD = 198$) in the clear condition, $z = 4.96$, $p < .001$, which can also support the clarity-valence congruency effect.

Similar with Experiment 1, negative words, in Experiment 2, were evaluated better in the blurry condition ($M = 603$ ms, $SD = 194$) compared to the clear condition ($M = 614$ ms, $SD = 165$), $z = 2.54$, $p = .011$. Positive words tended to be evaluated better in the clear condition ($M = 574$ ms, $SD = 143$) than in the blurry condition ($M = 583$ ms, $SD = 165$), though insignificant, $z = 1.25$, $p = .176$. Also, negative words ($M = 614$ ms, $SD = 165$) were evaluated more poorly relative to positive words ($M = 574$ ms, $SD = 143$) in the clear condition, $z = 6.06$, $p < .001$.

In Experiment 3, negative words were evaluated better in the blurry condition ($M = 634$ ms, $SD = 108$) compared to the clear condition ($M = 652$ ms, $SD = 119$), $z = 3.18$, $p = .001$, whereas the evaluation of positive words was better in the clear condition ($M = 574$ ms, $SD = 105$) than in the blurry condition ($M = 620$ ms, $SD = 118$), $z = 11.31$, $p < .001$.

In the valence-evaluation task in Experiment 5, the evaluation of positive words was better in the clear condition ($M = 926$ ms, $SD = 460$) than in the blurry condition ($M = 1138$ ms, $SD = 515$), $z = 7.04$, $p < .001$. Though the pairwise comparison between the Negative-Blurry and the Negative-Clear condition was not significant, $z = 0.31$, $p = .760$, the other two comparisons showed that negative words ($M = 1061$ ms, $SD = 572$) were evaluated better than positive words ($M = 1138$ ms, $SD = 515$) in the blurry condition, $z = 3.38$, $p < .001$, and negative words ($M = 1070$ ms, $SD = 548$) were evaluated more poorly relative to positive words ($M = 926$ ms, $SD = 460$) in the clear condition, $z = 4.92$, $p < .001$.

Taken together, these results are in line with those based on the standard ANOVA, suggesting that a performance (i.e., IES) advantage was shown in the congruent conditions (clear-positive, blurry-negative). The clarity-valence congruency effect and its unidirectional nature (i.e., existing in the concrete-to-abstract direction but not the reverse abstract-to-concrete direction) were supported.

REFERENCES

Ernst, M.D. (2004). Permutation Methods: A Basis for Exact Inference. *Statist. Sci.* 19(4)**,** 676-685. doi: 10.1214/088342304000000396.

Kabacoff, R. I. (2011). *R in Action*. New York: Manning Publications Co.

Morís Fernández, L., and Vadillo, M.A. (2019). Reaction times: Many ways of inadvertently obtaining a false positive. doi: 10.31219/osf.io/d4yqz.
